# Nonlinear association of fibrinogen levels with functional prognosis in patients with acute ischemic stroke: a prospective cohort study

**DOI:** 10.1186/s12883-024-03674-4

**Published:** 2024-05-20

**Authors:** Feng Chen, Yong Han, Haofei Hu, Yuying Guo, Zhe Deng, Dehong Liu

**Affiliations:** 1 College of Medicine, Shantou University, Shantou, Guangdong Province 515041 China; 2 Department of Emergency, Shenzhen Second People’s Hospital, The First Affiliated Hospital of Shenzhen University, No.3002 Sungang Road, Futian District, Shenzhen, Guangdong Province 518000 China; 3 Department of Nephrology, Shenzhen Second People’s Hospital, The First Affiliated Hospital of Shenzhen University, Shenzhen, Guangdong Province 518000 China

**Keywords:** Acute ischemic stroke, Prognosis, Nonlinear relationship, Fibrinogen, Modified Rankin scale score

## Abstract

**Objective:**

Fibrinogen, essential in primary hemostasis, platelet aggregation, and leukocyte-endothelial interactions, is also associated with a heightened risk of acute ischemic stroke (AIS). However, its influence on AIS patient outcomes is unclear. This study examines the correlation between fibrinogen levels and the risk of unfavorable outcomes three months post-AIS.

**Methods:**

This is a secondary analysis of a prospective cohort study conducted in Korea. The sample consisted of 1851 AIS patients who received treatment at a Korean hospital between January 2010 and December 2016. Statistical models were established to understand the relationship between fibrinogen levels(mg/dL) and unfavorable outcomes(mRs ≥ 3), including logistic regression models, Generalized Additive Models (GAM), and smooth curve fitting (penalized splines). The log-likelihood ratio test has been utilized to evaluate the best fit. To ensure the robustness of the results, sensitivity analyses were conducted by reanalyzing the relationship after excluding participants with TG > 200 mg/dl and BMI > 25 kg/m^2^. Subgroup analyses were also performed to assess whether influencing factors modify the association between fibrinogen levels and unfavorable outcomes.

**Results:**

After adjusting for multiple covariates including age, BMI, sex, LDL-c, TG, HGB, HDL-c, BUN, FPG, ALB, PLT, AF, hypertension, smoking, DM, mRs score at admission, the binary logistic regression model demonstrated revealed a significant positive association between fibrinogen levels and the risk of unfavorable outcomes in AIS patients (OR = 1.215, 95% CI: 1.032–1.429, *p* = 0.019). Sensitivity analyses supported these findings, with similar ORs observed in subsets of patients with TG < 200 mg/dL (OR = 1.221, 95% CI: 1.036–1.440) and BMI < 25 kg/m^2^ (OR = 1.259, 95% CI: 1.051–1.509). Additionally, the relationship between fibrinogen levels and outcomes was nonlinear, with a critical threshold of 2.74 g/L. Below the inflection point, the OR for unfavorable outcomes was 0.666 ((95% CI: 0.360, 1.233, *p* = 0.196), whereas above it, the OR increased to 1.374 (95% CI: 1.138, 1.659).

**Conclusions:**

This study has provided evidence of a positive and nonlinear correlation between fibrinogen levels and 3-month poor functional outcomes in patients with AIS. When fibrinogen levels exceeded 2.74 g/L, a significant and positive association was observed with the risk of poor outcomes. This study provides a further reference for optimizing rehabilitation exercises and facilitating clinical counseling in patients with acute ischemic stroke.

**Supplementary Information:**

The online version contains supplementary material available at 10.1186/s12883-024-03674-4.

## Introduction

Acute Ischemic Stroke (AIS) often leads to death or disability in patients [1–3]. Accurately predicting the prognosis of AIS patients is crucial for developing personalized treatment plans and improving patient outcomes [4]. Age, cardiac disease, diabetes, etiology of stroke, and hypertension have been identified as major prognostic factors for stroke [5–7]. Indeed, biomarkers play a key role in the management and prognostic assessment of AIS. Some biomarkers not only aid in diagnosis but can also predict disease progression and outcomes, providing clinicians with objective, quantifiable indicators to assist in clinical decision-making [8, 9].

Fibrinogen is an acute-phase protein that plays a crucial role in platelet activation, coagulation, and inflammatory responses [10–12]. In the general population, higher levels of fibrinogen have been identified as a risk factor for cardiovascular events, including ischemic stroke [13–15]. Research has confirmed that inflammatory responses and a hypercoagulable state are associated with poor prognosis following a stroke [16]. However, few studies have explored the relationship between fibrinogen levels and functional prognosis after AIS, with inconsistent findings reported. One study confirmed that hyperfibrinogenemia (> 4.0 g/L) is associated with a 1.76-fold increase in the risk of in-hospital mortality (hazard ratio, HR = 1.76; 95% confidence interval [CI]: 1.10–2.81), but found no significant association with poor prognosis at discharge (mRS > 3) [17]. A study from India demonstrated through regression analysis that fibrinogen levels (mg/dl) were positively correlated with early neurological deterioration (OR = 1.004, 95% CI 1.000-1.007, *P* = 0.038) [18]. Another study showed that after adjusting for potential confounders, the relationship between fibrinogen levels and poor outcomes after AIS (mRS > 3) was not significant (OR = 1.05, 95% CI: 0.90–1.21) [19]. However, it is well known that high levels of fibrinogen are considered a risk factor for ischemic stroke. Therefore, we propose a hypothesis that elevated fibrinogen may still be positively correlated with adverse outcomes in AIS patients. Additionally, considering the inconsistency of these results (based on linear regression), which may be influenced by non-linear associations and variations in the distribution range of fibrinogen, there may exist a non-linear relationship between fibrinogen levels and adverse outcomes in AIS patients. The objective of this study was to investigate the relationship, both linear and nonlinear, between fibrinogen levels and unfavorable outcomes in AIS patients, utilizing data from a Korean cohort study.

## Methods

### Study design

This is a secondary analysis based on a prospective cohort study in South Korea [20]. The original researchers utilized prospective single-center registry data to collect information on patients diagnosed with AIS admitted within one week of symptom onset, covering January 2010 to December 2016 [20]. Upon admission, professional nurses measured patients’ height and weight using an automatic weighing scale (Model GL-150, G-Tech International, Hwaseong-si, Gyeonggi-do, South Korea). For severely affected stroke patients who could not stand independently, weight was measured using a bed scale and height with a tape measure. Laboratory data, including Scr, TG, and fibrinogen were extracted from electronic medical records. A modified Rankin Scale (mRs) score was also conducted upon admission to assess the initial neurological condition [20].

### Data source and study population

This second analysis employed data and information from the article “Geriatric Nutritional Risk Index Predicts Poor Outcomes in Patients with Acute Ischemic Stroke - Automated Undernutrition Screen Tooll” by Kang MK, et al., published under the Creative Commons Attribution License allowing unlimited use with proper attribution [20]. Originally approved by Seoul National University Hospital’s IRB (IRB number 1009-062-332) with a patient consent waiver, no additional ethical approval was needed for this second analysis [20].

The initial study initially included 2,084 patients diagnosed with acute AIS. However, 72 patients lacking dysphagia testing or laboratory information within 24 h of admission, along with 106 individuals without a three-month modified Rankin Scale (mRS) score after discharge, were subsequently excluded. As a result, the final sample size for the original study amounted to 1,906 participants. For the current study, an additional exclusion criterion was applied, resulting in the exclusion of participants with fibrinogen deficiency (*n* = 22) as well as those with fibrinogen abnormalities and extreme values (greater than or less than three standard deviations from the mean) (*n* = 33).

### Variables

#### Fibrinogen

Fibrinogen, measured in g/L, is considered a continuous variable for patients with AIS within 24 h of admission [20]. Fibrinogen was transformed into a categorical variable based on quartiles: Q1 (< 2.80), Q2 (2.80–3.19), Q3 (3.19–3.67), and Q4 (≥ 3.67).

#### Neurological recovery status of acute ischemic stroke patients at three months

The neurological recovery status of AIS patients at three months was evaluated using the mRS score [21]. Data was collected through telephone interviews or structured outpatient interviews [20]. An unfavorable outcome was defined as an mRS score of 3 or higher [20, 21].

#### Covariates

In our study, we selected covariates based on previous research and our clinical expertise [20, 22–24]. The following variables were utilized as covariates: (i) Categorical variables: sex, diabetes mellitus (DM), age, atrial fibrillation (AF), smoking, and hypertension; (ii) continuous variables: hemoglobin concentration (HGB), serum high-density lipoprotein cholesterol (HDL-c), body mass index (BMI), total serum cholesterol (TC), geriatric nutritional index(NRI), fasting blood glucose (FBG), blood Urea Nitrogen (BUN), serum low-density lipoproteins cholesterol (LDL-c), platelet (PLT), mRs score at admission, serum triglyceride (TG), international normalized ratio(INR), serum albumin (ALB), activated partial thromboplastin time(APTT).

#### Missing data processing

In our study, a total of 1 (0.05%), 1 (0.05%), 67 (3.62%), 91 (4.92%), 99 (5.35%), and 130 (7.02%) participants had missing data for WBC, TC, LDL-c, HDL-c, TG, and FBG, respectively. To address this issue and minimize potential bias in the modeling phase, we employed multiple imputation techniques to estimate the missing values [25, 26]. Age, LDL-c, HGB, sex, TG, ALB, PLT, BUN, FBG, hypertension, DM, AF, smoking, and mRs scores at admission were included in the estimation model (number of iterations was 10, and regression type was linear regression). The missing data analysis process used the missing at random (MAR) assumption [25].

### Statistical analysis

Mean and standard deviation are reported for normally distributed continuous variables, while medians (quartiles) are reported for skewed distributions, and frequencies with percentages are reported for categorical variables. Differences among different fibrinogen quartile groups were tested using the χ2 test, one-way ANOVA test, or Kruskal-Wallis H test, depending on the type of variable.

Two different models were constructed using univariate and multivariate binary logistic regression (binary dependent variable: mRS < 3; mRS ≥ 3) and linear regression analysis (mRS as a continuous variable) to investigate the association between fibrinogen and the risk of unfavorable outcomes after AIS. The variables adjusted in the multivariable logistic regression model include sex, HDL-c, age, BMI, LDL-c, HGB, PLT, TG, ALB, BUN, hypertension, DM, smoking, atrial fibrillation, and mRS score at admission. Odds ratios (OR) and their corresponding 95% confidence intervals (95% CI) were calculated and reported. Adjustment for confounding variables was based on clinical knowledge, existing literature, and results from univariate analyses. The multivariate regression equation adjusted for variables excluding TC because of its covariance with other factors (Supplementary Table S1). Besides, the decision to adjust for covariates is also guided by the following principle: when a covariate is added to the model, the matched odds ratio changes by at least 10% (Supplementary Table S2) [27].

The nonlinear relationship between fibrinogen and adverse outcomes was further investigated through generalized additive modeling (GAM) and smooth curve fitting using penalized splines. When nonlinearity was observed, an iterative algorithm was employed to compute inflection points, which were subsequently used to construct binary logistic regression models on each side of the inflection point. A log-likelihood ratio test was utilized to determine the most appropriate model for describing the relationship between fibrinogen levels and unfavorable prognosis in AIS patients.

A set of sensitivity analyses were performed to assess the robustness of the findings. Fibrinogen was transformed into a categorical variable using quartiles, and *P* values for trends were computed to evaluate the impact of fibrinogen as a continuous variable and to investigate potential nonlinearity. Given the significant correlation between obesity, hypertriglyceridemia, and unfavorable prognosis in patients with AIS [25, 28]. As a result, we excluded patients with TG > 200 mg/dL and BMI ≥ 25 kg/m^2^ from other sensitivity analyses to investigate the association between fibrinogen and unfavorable outcomes in AIS patients [29, 30]. Besides, participants were divided into high fibrinogen and non-high fibrinogen groups based on the clinical cutoff value (4.0 g/L) for propensity score(PS) matching analysis [31]. Fibrinogen was used as the independent variable, and all baseline parameters listed in Supplementary Table S3 were used as variables in the non-fitted multivariate logistic regression model to calculate PS. Variables used for matching included sex, DM, age, AF, smoking, hypertension, HDL-c, BMI, TC, FPG, BUN, LDL-c, PLT, INR, mRs score at admission, TG, ALB, GLB, and APTT, using a 1:1 matching scheme without replacement (greedy matching algorithm) with a caliper width of 0.05. Additionally, potential unobserved confounders between fibrinogen and poor outcome were investigated by calculating E-value [32]. Furthermore, we used generalized additive modeling (GAM) to incorporate continuous covariates as curves in the equations to ensure the robustness of the results.

Besides, subgroup analyses on various subgroups, including sex, age, hypertensive disease, diabetes, and smoking status, using stratified binary logistic regression models. Age was categorized into < 60, 60–70, 70–80, and ≥ 80 years [7]. Adjustments were made for each stratification factor, such as sex, age, HDL-c, BMI, LDL-c, HGB, PLT, TG, ALB, BUN, hypertension, DM, smoking, AF, and mRs scores at admission. Moreover, likelihood ratio tests were performed to compare models with and without interaction terms.

The STROBE statement was adhered to when documenting all findings [27]. Statistical analysis was performed using the R software (http://www.R-project.org, The R Foundation) and the EmpowerStats software (http://www.empowerstats.com, X&Y Solutions, Inc, Boston, MA). Statistical significance was determined by considering two-sided *P* values less than 0.05.

## Results

**Fig. 1:**
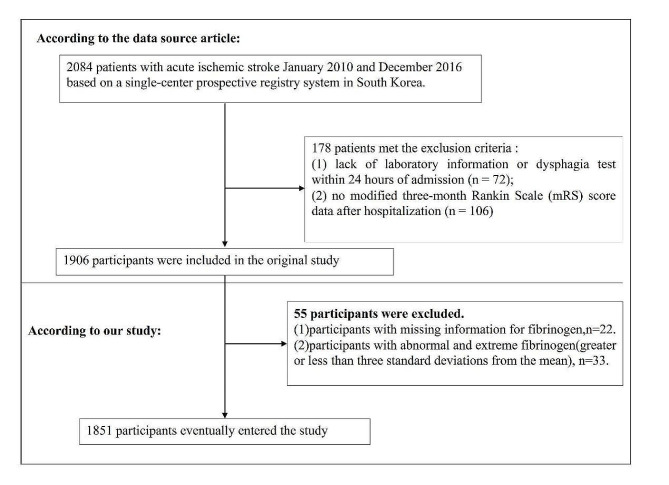
Flowchart of study participants

A total of 1851 participants were included in this second analysis, consisting of 1,134 males and 717 females. Figure 1 depicted the participant selection process. The number of participants aged < 60, 60–70, 70–80, and ≥ 80 years were 426, 487, 651, and 287, respectively. The prevalence of hypertension, DM, and AF was 63.1%, 31.87%, and 21.56%, respectively. Fibrinogen levels are normally distributed, ranging from 1.25 to 5.85 g/L, with a mean (standard deviation) of 3.282 ± 0.782 g/L (Supplementary Figure S1). Table 1 listed the demographic and clinical characteristics of the study participants. The participants were divided into subgroups based on fibrinogen quartiles(< 2.80, 2.8–3.19, 3.19–3.67, ≥ 3.67 g/L). Compared to the first quartile group (fibrinogen < 2.8 g/L), patients in the fourth quartile had higher levels of PLT, LDL-c, BUN, Scr, APTT, and mRS scores at admission and lower levels of NRI and HDL-c. Additionally, participants in the fourth quartile had a higher percentage of females, hypertension, DM, and were ≥ 80 years of age compared to those in the first quartile.

**Table 1:** The baseline characteristics of participants

Fibrinogen quartile(g/L)	Q1(< 2.80)	Q2(2.8–3.19)	Q3(3.19–3.67)	Q4(≥ 3.67)	*P*-value
participants	459	460	459	473	
HGB(g/L)	135.54 ± 20.03	136.76 ± 17.88	137.57 ± 18.62	131.19 ± 21.56	< 0.001
PLT (10*10^9^/L)	203.81 ± 62.18	214.40 ± 64.29	230.08 ± 67.10	241.59 ± 79.19	< 0.001
TC (mg/dL)	175.59 ± 38.12	179.55 ± 41.50	185.89 ± 42.91	178.34 ± 50.00	0.003
TG (mg/dL)	96.00 (75.00-131.00)	98.00 (74.00-133.00)	97.00 (73.00-140.50)	95.00 (75.00-128.00)	0.853
HDL-c(mg/dL)	48.36 ± 13.76	48.12 ± 13.43	47.91 ± 13.16	43.75 ± 13.24	< 0.001
LDL-c(mg/dL)	103.92 ± 32.88	106.82 ± 36.71	112.59 ± 36.66	108.36 ± 42.23	0.005
BUN(mg/dl)	16.41 ± 7.34	16.83 ± 6.35	17.25 ± 8.36	19.38 ± 11.08	< 0.001
Scr(mg/dl)	0.89 (0.73–1.04)	0.85 (0.73–1.04)	0.90 (0.73–1.10)	0.91 (0.76–1.16)	< 0.001
NRI	105.72 ± 9.12	106.90 ± 8.93	106.75 ± 9.03	102.36 ± 10.22	< 0.001
ALB(g/dL)	4.07 ± 0.39	4.11 ± 0.37	4.09 ± 0.37	3.87 ± 0.46	< 0.001
GLB(g/dL)	6.98 ± 0.60	7.05 ± 0.56	7.11 ± 0.57	6.96 ± 0.64	< 0.001
FBG (mg/dl)	104.59 ± 38.12	106.08 ± 36.19	108.46 ± 39.81	109.97 ± 40.50	0.147
INR	1.04 ± 0.24	1.05 ± 0.31	1.03 ± 0.26	1.07 ± 0.42	0.290
APTT (ms)	30.75 ± 5.35	30.61 ± 4.48	31.50 ± 6.03	31.36 ± 4.79	0.018
BMI (kg/m^2^)	23.38 ± 3.05	23.68 ± 3.23	23.79 ± 3.36	23.23 ± 3.35	0.029
mRS score at admission	0.59 ± 0.20	0.62 ± 0.27	0.65 ± 0.23	0.75 ± 0.42	0.209
Sex					0.327
Men	295 (64.27%)	271 (58.91%)	274 (59.69%)	294 (62.16%)	
Women	164 (35.73%)	189 (41.09%)	185 (40.31%)	179 (37.84%)	
Age(years)					0.041
<60	128 (27.89%)	102 (22.17%)	101 (22.00%)	95 (20.08%)	
60–70	117 (25.49%)	120 (26.09%)	128 (27.89%)	122 (25.79%)	
70–80	164 (35.73%)	165 (35.87%)	154 (33.55%)	168 (35.52%)	
≥ 80	50 (10.89%)	73 (15.87%)	76 (16.56%)	88 (18.60%)	
Hypertension	265 (57.73%)	283 (61.52%)	311 (67.76%)	309 (65.33%)	0.010
Diabetes	124 (27.02%)	132 (28.70%)	154 (33.55%)	180 (38.05%)	0.001
Smoking	179 (39.00%)	159 (34.57%)	195 (42.48%)	198 (41.86%)	0.056
Atrial fibrillation	107 (23.31%)	95 (20.65%)	94 (20.48%)	103 (21.78%)	0.709

Values are mean ± standard deviation or median (quartile) or number (%)

TC: total cholesterol; PLT: platelet; HGB: hemoglobin concentration; LDL-c: low-density lipoproteins cholesterol; GLB: globulin; HDL-c: high-density lipoprotein cholesterol; BUN: blood urea nitrogen; Scr: serum creatinine; NRI: nutritional risk index; TG: triglyceride; FBG: fasting blood glucose; ALB: serum albumin; BMI: body mass index; mRS: modified Rankin scale;

### Incidence of unfavorable prognosis three months after acute ischemic stroke

Table 2 presented data indicating that a total of 526 participants exhibited an unfavorable prognosis. The overall incidence rate of adverse outcomes was 28.42%. Specifically, the incidence rates of unfavorable prognosis among participants in the first to fourth quartiles of fibrinogen were 24.18%, 24.35%, 27.89%, and 37.00%, respectively. When stratified by age groups (< 60, 60 to < 70, 70 to < 80, and ≥ 80), the incidence of unfavorable outcomes in individuals with AIS was higher in females compared to males, regardless of age category. Additionally, the incidence of unfavorable outcomes was observed to increase with age in both males and females (Supplementary Figure S2).

**Table 2:** Incidence rate of unfavorable outcomes 3 months after stroke

Fibrinogen	Participants(*n*)	unfavorable outcome events(mRS score ≥ 3)	Incidence of unfavorable outcome (95% CI) (%)
Total	1851	526	28.42(26.1–30.3)
Q1	436	111	24.18(28.4–37.2)
Q2	443	112	24.35(26.0-34.5)
Q3	442	128	27.89(21.5–29.6)
Q4	443	175	37.00 (20.4–28.4)
P for trend			< 0.001

mRS: modified rankin scale; FIB: fibrinogen

### Results of univariate analysis using binary logistic regression models

The results of the univariate analysis indicate significant associations between certain factors and the risk of unfavorable outcomes in AIS patients. Specifically, BMI、HGB、TG、ALB, NRI, and LDL-c are negatively correlated with the risk. Conversely, BUN, mRs score at admission, fibrinogen, and FBG positively correlate with unfavorable outcome risks. Additionally, individuals aged 80 years or older, females, patients with hypertension, and those with DM are more likely to experience unfavorable outcomes (all *P* < 0.05). (Supplementary Table S4).

### Results of the multivariate analysis using binary logistic regression and linear regression models

Firstly, when treating the dependent variable as a binary outcome (unfavorable outcome versus non-unfavorable outcome), multivariate logistic regression analysis revealed that each 1 g/L increase in fibrinogen was associated with a 22.9% increase in the incidence of unfavorable outcomes in AIS patients (OR = 1.229, 95% CI: 1.054, 1.434; *p* = 0.008). The variables adjusted for included sex, HDL-c, age, BMI, LDL-c, HGB, PLT, TG, ALB, Scr, hypertension, DM, smoking, AF, and mRS score at admission (Table 3 Model I). When fibrinogen was analyzed categorically, the test for trend in effect size (OR values) was statistically significant (P for trend < 0.05). These results indicate that the trend in effect size between groups when fibrinogen levels are transformed into categorical variables is consistent with the results when fibrinogen is treated as a continuous variable (Table 3 Model I).

Besides, when using the 3-month post-AIS mRS score as the outcome variable, multivariate logistic regression analysis(adjusted for sex, HDL-c, age, BMI, LDL-c, HGB, PLT, TG, ALB, BUN, hypertension, DM, smoking, AF, and the mRS score at admission) showed that each 1 g/L increase in fibrinogen is associated with an increase of 0.163 points in the 3-month post-AIS mRs score (β = 0.163, 95% CI: 0.058, 0.268; *P* = 0.002) (Supplementary Table S5). When fibrinogen levels were categorized, the trend in effect size(OR value) between groups remained consistent with the results when fibrinogen was treated as a continuous variable.

**Table 3:** Relationship between fibrinogen and unfavorable outcomes 3-month(mRS ≥ 3) after acute ischemic stroke in different models

Exposure	Non-AIS(*n* = 1325)	AIS(*n* = 526)	Model I(OR 95%CI)	Model II (OR 95%CI)
Fibrinogen (g/L)	3.23 ± 0.68	3.43 ± 0.83	1.229 (1.054, 1.434) 0.008	1.201 (1.019, 1.415) 0.029
Fibrinogen quartiles				
Q1	348 (26.26%)	111 (21.10%)	Ref	Ref
Q1	348 (26.26%)	112 (21.29%)	0.977 (0.703, 1.358) 0.891	0.990 (0.707, 1.387) 0.954
Q3	331 (24.98%)	128 (24.33%)	1.164 (0.842, 1.609) 0.359	1.129 (0.809, 1.575) 0.475
Q4	298 (22.49%)	175 (33.27%)	1.379 (1.003, 1.897) 0.048	1.339 (0.956, 1.876) 0.089
P for trend			< 0.001	< 0.001

Model I: we adjusted age, BMI, sex, LDL-c, TG, HGB, HDL-c, BUN, FPG, ALB, PLT, AF, hypertension, smoking, DM, mRs score at admission

Model II: we adjusted age, sex, BMI(smooth), LDL-c(smooth), HGB(smooth), TG(smooth), HDL-c(smooth), BUN(smooth), FBG(smooth), ALB(smooth), PLT(smooth), AF, hypertension, smoking, DM, mRs score at admission(smooth)

OR: odds ratios; CI: confidence; Ref: reference

### Sensitivity analyses

A series of sensitivity analyses were conducted to verify the reliability of the results. Firstly, a GAM was used to include continuous covariates as curves in the equation; the results from Model II in Table 3 are consistent with those from the fully adjusted Model I. Additionally, an E-value was calculated to assess the susceptibility of the study results to unmeasured confounding factors. Additionally, an E-value of 1.44, surpassing the relative risk of 1.32 for unmeasured confounders, suggests minimal impact from unmeasured or unknown confounders on the fibrinogen-unfavorable outcome relationship. Besides, other sensitivity analyses that excluded participants with a BMI ≥ 25 kg/m^2^ and TG ≥ 200 mg/dL also yielded similar results, showing a positive correlation between fibrinogen levels and the risk of unfavorable outcomes at 3 months in AIS patients. (Supplementary Table S6).

Additionally, a propensity score matching analysis involved one-to-one matching of 262 AIS patients with high fibrinogen levels to 262 AIS patients with non-high fibrinogen levels. After matching, there were no significant differences in baseline characteristics between the high and non-high fibrinogen groups (Supplementary Table S3). Further logistic regression analysis indicated that AIS patients with high fibrinogen levels had a 68.1% higher risk of unfavorable outcomes than those with lower fibrinogen levels (Supplementary Table S7).

### Generalized additive models address the nonlinear relationship between fibrinogen and unfavorable outcomes

**Fig. 2:**
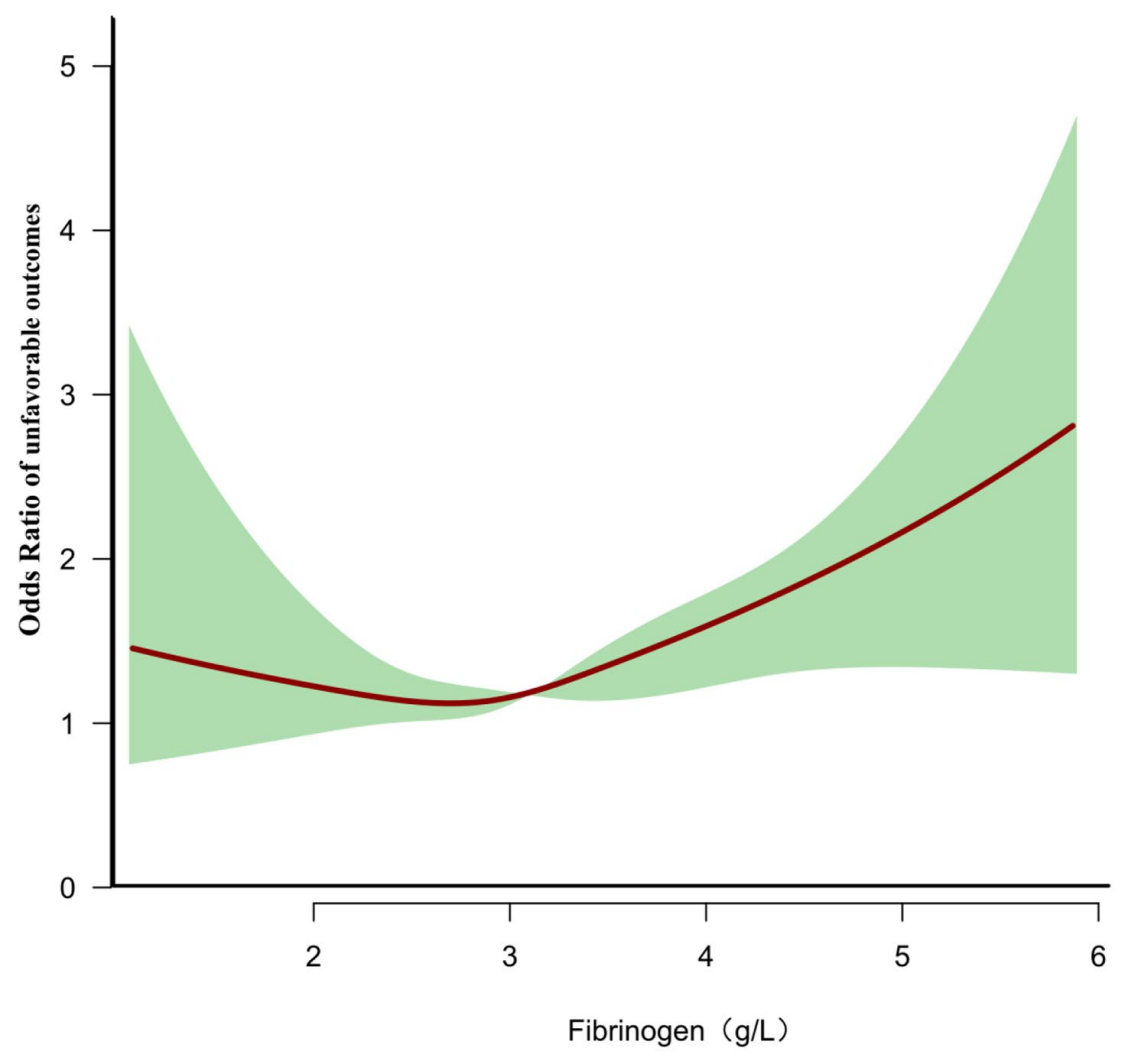
The nonlinear relationship between fibrinogen and the risk of unfavorable outcomes in patients with acute ischemic stroke. A nonlinear relationship was detected after adjusting for age, sex, BMI, LDL-c, HGB, TG, HDL-c, BUN, FPG, ALB, PLT, AF, hypertension, smoking, DM, and mRs score at admission

Using GAM and smooth curve fitting while adjusting for various covariates, including sex, age, HDL-c, BMI, LDL-c, HGB, PLT, TG, ALB, BUN, hypertension, DM, smoking, AF, and mRs scores at admission, we observed a nonlinear association between fibrinogen levels in patients with AIS and the risk of unfavorable outcomes(P for log-likelihood ratio test = 0.047) (Fig. 2). The inflection point of fibrinogen level (2.74 g/L) was determined using a recursive method. Subsequently, a two-piece logistic regression model was employed to estimate the OR and CI for both sides of the inflection point. The OR for the left side of the inflection point was 0.666 (95% CI: 0.360, 1.233, *P* = 0.196), indicating no statistically significant difference. In contrast, the OR for the right side of the inflection point was 1.374(95% CI: 0.360, 1.233, *p* < 0.001) (Table 4).

**Table 4:** Relationship between fibrinogen and unfavorable outcome 3-month after acute ischemic stroke analyzed by two-piecewise linear regression model

Outcome:	OR (95%CI) *P*
Fitting model by two-piecewise linear regression	
Inflection point of fibrinogen (g/L)	2.74
≤ Inflection point	0.666 (0.360, 1.233) 0.196
> Inflection point	1.374 (1.138, 1.659) < 0.001
P for log-likelihood ratio test	0.047

Adjusted age, sex, BMI, LDL-c, HGB, TG, HDL-c, BUN, FPG, ALB, PLT, AF, hypertension, smoking, DM, mRs score at admission

AIS: acute ischemic stroke. OR: odds ratios; CI: confidence; Ref: reference

### Results of subgroup analyses

In all prespecified or exploratory subgroup assessments (Supplementary Table S8), age, sex, smoking status, DM, and hypertension did not significantly interact with fibrinogen. These influences did not modify or alter the relationship between fibrinogen and unfavorable outcomes in patients with AIS.

## Discussion

The objective of this study was to examine the correlation between fibrinogen and the neurological status of patients with AIS over a period of three months. The findings revealed a positive association between fibrinogen and the risk of unfavorable outcomes after AIS. Furthermore, a threshold effect curve with an inflection point for fibrinogen of 2.74 g/L was observed. Distinct relationships between fibrinogen and unfavorable outcomes were observed on either side of this inflection point.

Fibrinogen plays a crucial role in primary hemostasis, platelet aggregation, and leukocyte-endothelial cell interactions. Additionally, it serves as a significant determinant of blood viscosity and functions as an acute-phase protein [33]. Previous research has consistently demonstrated a strong association between fibrinogen and an elevated risk of ischemic stroke [13, 14, 34–36]. Therefore, we hypothesize that fibrinogen may also be associated with an increased risk of poor prognosis in ischemic stroke. Nevertheless, there is a paucity of research on the impact of fibrinogen on the prognosis of patients with AIS, and the conclusions are inconsistent. Several studies with limited sample sizes have indicated that elevated fibrinogen levels are linked to higher mortality rates among individuals experiencing acute stroke [37–39]. One study found that elevated fibrinogen levels at 24 h after rt-PA treatment were associated with an approximately 40% increase in mortality at 90 days [40]. A study using data from China’s National Stroke Registry III, which included 10,518 patients with AIS within seven days of onset (baseline), showed that high baseline fibrinogen levels were associated with poor functional prognosis (mRs > 2) (OR = 1.63; 95%=CI:1.35–1.97) [41]. Similar results were obtained in a small-sample study from India, where baseline fibrinogen (per 1 mg/dL) was positively associated with poor functional prognosis (OR = 1.011, 95% CI 1.006–1.015, *P* < 0.001) [18]. However, some studies have yielded inconsistent results. A previous study showed that fibrinogen levels were associated with poor functional prognosis after adjustment for simple risk factors, but this association disappeared after further adjustment for underlying factors [19]. In another earlier study, which included 83 patients treated with intravenous alteplase, fibrinogen levels at admission were not associated with prognosis (mRS > 2) at 3 months [42]. There are several possible reasons for the inconsistency of the findings. First, there were differences in the populations and sample sizes of these studies. Second, the outcome variables were different. Third, the adjusted variables were different. The results of our study are consistent with previous findings that fibrinogen levels are positively associated with poor outcomes in AIS. Besides, we also found that baseline fibrinogen levels are positively correlated with the mRS scores at 3 months post-AIS. In addition, this study explored the relationship between fibrinogen and adverse outcomes by using fibrinogen as both a categorical and continuous variable, thereby minimizing information loss and quantifying the relationship. The sensitivity analyses also focused on participants with BMI < 25 kg/m^2^ or TG < 200 mg/dL, and the results further confirmed the existence of the relationship in this group of participants, validating the stability of the results. In conclusion, these results suggest that fibrinogen may be a useful marker for predicting the prognosis of acute ischemic stroke, providing a reference for optimizing the rehabilitation of stroke patients and facilitating doctor-patient communication.

The underlying mechanisms by which fibrinogen levels play a role in prognosis have not been well elucidated. Blood fibrinogen levels reflect inflammatory states and coagulation disorders. Both inflammatory response and hypercoagulability are associated with poor prognosis [16, 43].

Additionally, our study is the first to observe a nonlinear relationship between fibrinogen levels and the risk of unfavorable outcomes in AIS patients. When fibrinogen levels exceed 2.74 g/L, there is a significant positive association with the incidence of unfavorable outcomes three months after AIS. This provides a reference for optimizing the rehabilitation of AIS patients in clinical settings. When a patient’s fibrinogen level exceeds 2.74 g/L, it indicates a potentially poorer prognosis, with higher levels correlating with worse outcomes. These patients may require better rehabilitation and care to minimize the risk of unfavorable outcomes.

Our study possesses several strengths. Firstly, we utilized both quartiles of fibrinogen as a categorical variable and fibrinogen as a continuous variable to examine its association with unfavorable outcomes in AIS patients. This approach reduces information loss and quantifies the relationship effectively. Secondly, addressing the nonlinear relationship represents a significant advancement compared to prior research. Thirdly, we employed the multiple imputation method to handle missing data, enhancing statistical power and mitigating potential bias resulting from missing covariate information. Moreover, to ensure the reliability of our findings, we conducted a series of sensitivity analyses. These included transforming the independent variable, calculating E-values to explore the possibility of unmeasured confounders, and reanalyzing the association between fibrinogen and unfavorable outcomes in AIS after excluding participants with a BMI ≥ 25 kg/m^2^ and TG ≥ 200 mg/dl. Additionally, a propensity-matched analysis was conducted to assess the relationship between high fibrinogen levels(≥ 4.0 g/L) and unfavorable outcomes in AIS patients.

It is important to acknowledge potential limitations in our study. Firstly, our study focused on Korean subjects, and further validation is necessary to assess the generalizability of these results to other racial groups. Secondly, fibrinogen measurements were conducted solely at the time of admission and were not repeated subsequently. Consequently, we lack information on whether fibrinogen levels changed within the first 24 h after admission, which warrants further investigation. Thirdly, this study’s outcome measure, the mRS score at three months post-AIS, lacks inclusion of the Glasgow Outcome Scale-Extended (GOSE) or assessments of long-term functional outcomes. While the mRS is widely used for evaluating post-stroke function, it does not fully capture all aspects of patient recovery. Future research should extend follow-up durations and include broader assessment tools like the GOSE to more thoroughly evaluate patient outcomes. Fourthly, some variables had incomplete data. For instance, the original study database provided age-stratification information in terms of ten intervals rather than the specific ages of patients, potentially resulting in incomplete data on certain variables. Future study designs may benefit from collecting more detailed variable information. Fifth, as with all observational studies, the presence of uncontrolled or unmeasured confounding factors remains a possibility, even when known potential confounders have been accounted for. Additionally, this study relied on secondary analysis of published data, which limited adjustments for variables not included in the dataset, such as information on intravenous thrombolysis or endovascular thrombectomy, antihyperglycemic medications, onset-to-door time (ODT), and timing of head CT scans. Although we calculated E-values to quantify the potential effects of unmeasured confounders, they are not a complete substitute for the effects of these confounders. Moving forward, we will initiate new study to collect more variable information.

## Conclusion

This study revealed a positive and nonlinear relationship between fibrinogen levels and unfavorable functional outcomes in patients with AIS. When fibrinogen was ≥ 2.74 g/L, it was significantly and positively associated with poor function in patients with acute ischemic stroke. When the patient’s fibrinogen was greater than 2.74 g/L, it suggested that the patient’s prognosis might be poor, and this group of patients needed to enhance rehabilitation and nursing care to minimize the risk of poor prognosis, which provided a reference for clinical optimization of rehabilitation for patients with AIS.

### Electronic supplementary material

Below is the link to the electronic supplementary material.

Supplementary Material 1

## Data Availability

Users can download data from the “PLos one” database, which can be reached at https://journals.plos.org/plosone/.

## Abbreviations

AIS: Acute ischemic stroke
ALB: Serum albumin
BMI: Body mass index
BUN: Blood urea nitrogen
CI: Confidence interval
DM: Diabetes mellitus
FBG: Fasting blood glucose
FIB: Fibrinogen
GAM: Generalized additive models
HDL-c: High-density lipoprotein cholesterol
HGB: Hemoglobin concentration
LDL-c: Low-density lipoproteins cholesterol
mRS: Modified Rankin Scale
OR: Odds ratio
PLT: Platelet
Scr: Serum creatinine
TC: Total cholesterol
TG: Triglyceride
