# Supplementary material for: Nonlinear association of fibrinogen levels with functional prognosis in patients with acute ischemic stroke: a prospective cohort study
Source: BMC Neurol. 2024 May 20;24:163. doi: 10.1186/s12883-024-03674-4 (PMC11103930; doi:10.1186/s12883-024-03674-4)
Supplement: Supplementary file 1 — Supplementary Material 1 [file 12883_2024_3674_MOESM1_ESM.docx]

**Nonlinear association of fibrinogen levels with functional prognosis in patients with** **acute ischemic stroke: a prospective cohort study**

**Running title: Fibrinogen** and **acute ischemic stroke**

Feng Chen^1.2#^, Yong Han^2#^ , Haofei Hu ^3#^, Yuying Guo ^2#^, Zhe Deng^2*^, Dehong Liu^2*^.

^1^ College of Medicine, Shantou University, Shantou 515041, Guangdong Province, China.

^2^ Department of Emergency, Shenzhen Second People's Hospital, The First Affiliated Hospital of Shenzhen University, Shenzhen 518000, Guangdong Province, China.

^3^ Department of Nephrology, Shenzhen Second People's Hospital, The First Affiliated Hospital of Shenzhen University, Shenzhen 518000, Guangdong Province, China.

^#^ Feng Chen, Yong Han, Haofei Hu, and Yuying Guo have contributed equally to this work.

***Corresponding author**

Zhe Deng

Department of Emergency,

Shenzhen Second People’s Hospital,

No.3002 Sungang Road, Futian District,

Shenzhen 518000,

Guangdong Province,

China

zdeng_emergency@126.com

***Corresponding author**

Dehong Liu

Department of Emergency,

Shenzhen Second People’s Hospital,

No.3002 Sungang Road, Futian District,

Shenzhen 518000,

Guangdong Province,

China

Dhliu_emergency@163.com

**Table S1 collinearity screening**

|  |  | Step 1 | Step 2 |
| --- | --- | --- | --- |
| Fibrinogen(g/L) |  | 1.2 | 1.2 |
| TC (mg/dl) |  | 6.8 | NA |
| HDL-c(mg/dl) |  | 1.7 | 1.3 |
| BMI (kg/m^2^) |  | 1.3 | 1.3 |
| HGB（g/dL |  | 1.9 | 1.8 |
| TG (mg/dl) |  | 1.6 | 1.3 |
| LDL-c(mg/dl) |  | 5.4 | 1.2 |
| ALB(g/dL) |  | 1.5 | 1.5 |
| GLB(g/dL) |  | 2.0 | 2.0 |
| BUN (mg/dl) |  | 1.9 | 1.9 |
| Scr (mg/dl) |  | 1.9 | 1.9 |
| Sex |  | 1.8 | 1.8 |
| Diabetes |  | 1.4 | 1.4 |
| Hypertension |  | 1.1 | 1.1 |
| PLT（10*109/L） |  | 1.2 | 1.2 |
| Age |  | 1.3 | 1.3 |
| mRS score at admission |  | 1 | 1 |
| Smoking |  | 1.5 | 1.5 |
| AF |  | 1.1 | 1.1 |
| FBG |  | 1.4 | 1.4 |

NA was the excluded variable

HGB, hemoglobin concentration; PLT, platelet; TC, total cholesterol; TG, triglyceride; LDL-c, low-density lipoproteins cholesterol; HDL-c, high-density lipoprotein cholesterol; FBG, fasting blood glucose; Scr, serum creatinine; BUN, blood urea nitrogen; GLB, globulin; ALB, serum albumin; BMI, body mass index; CHD, coronary heart disease; mRS, modified Rankin scale

**Table S2 Covariate Screening**

|  |  | basic model | full model | Selected covariates |
| --- | --- | --- | --- | --- |
| covariate | +/- term | FIB | FIB | BMI, HGB, TG, LDL-c, ALB, BUN, Sex, DM HT, Age, mRS at admission, smoking, AF,FBG |
|  | Starting regression coefficient | 0.3690 | 0.1950 |  |
| HDL-c | HDL | 0.3731 | 0.1877 |  |
| BMI | BMI | 0.3564 | 0.1985 |  |
| HGB | HB | 0.3177 * | 0.1914 |  |
| TG | TG | 0.3695 | 0.2023 |  |
| LDL-c | LDL | 0.3750 | 0.1959 |  |
| ALB | ALB | 0.2067 * | 0.3044 |  |
| BUN | BUN | 0.3412 | 0.1944 |  |
| GLB | GLB | 0.3574 | 0.1950 |  |
| Scr | Scr | 0.3706 | 0.1912 |  |
| Sex | factor(Sex) | 0.3703 | 0.2004 |  |
| DM | factor(DM) | 0.3542 | 0.1947 |  |
| Hypertension | factor(Hypertension) | 0.3624 | 0.1988 |  |
| PLT | PLT | 0.4036 | 0.2041 |  |
| Age | factor(Age) | 0.3357 | 0.2138 |  |
| mRS at admission | mRS at admission | 0.3615 | 0.1880 |  |
| Smoking | factor(Smoking) | 0.3816 | 0.1905 |  |
| AF | factor(AF) | 0.3766 | 0.1943 |  |
| FBG | FBG | 0.3550 | 0.2087 |  |

HGB, hemoglobin concentration; PLT, platelet; TC, total cholesterol; TG, triglyceride; LDL-c, low-density lipoproteins cholesterol; HDL-c, high-density lipoprotein cholesterol; FBG, fasting blood glucose; GLB, globulin; Scr, serum creatinine; BUN, blood urea nitrogen; ALB, serum albumin; BMI, body mass index; FIB, fibrinogen; mRS, modified Rankin scale

**Table S3 Baseline characteristics before and after propensity score matching.**

|  | Before matching | |  |  | After matching | |  |
| --- | --- | --- | --- | --- | --- | --- | --- |
| Fibrinogen quartile（g/L） | <4.0 | ≥4.0 | P-value |  | <4.0 | ≥4.0 | P value |
| participants | 1580 | 271 |  |  | 262 | 262 |  |
| HGB(g/L) | 13.63 ± 1.89 | 12.90 ± 2.30 | <0.001 |  | 13.04 ± 2.25 | 13.00 ± 2.26 | 0.8726 |
| PLT (10*10^9^/L) | 218.57 ± 65.98 | 246.18 ± 86.69 | <0.001 |  | 227.32 ± 89.19 | 240.53 ± 79.12 | 0.0737 |
| TC (mg/dL) | 180.38 ± 41.64 | 176.65 ± 53.19 | 0.192 |  | 177.49 ± 42.87 | 178.24 ± 53.23 | 0.8594 |
| TG (mg/dL) | 111.68 ± 58.22 | 105.04 ± 52.72 | 0.116 |  | 110.08 ± 65.94 | 105.42 ± 53.42 | 0.3745 |
| HDL-c(mg/dL) | 47.62 ± 13.38 | 43.46 ± 13.82 | <0.001 |  | 45.98 ± 13.49 | 43.96 ± 13.71 | 0.0895 |
| LDL-c(mg/dL) | 108.26 ± 36.22 | 105.98 ± 43.74 | 0.354 |  | 105.22 ± 37.56 | 106.90 ± 44.01 | 0.6387 |
| BUN(mg/dl) | 16.86 ± 7.41 | 21.08 ± 12.88 | <0.001 |  | 19.44 ± 11.05 | 20.95 ± 12.82 | 0.1479 |
| Scr(mg/dl) | 1.02 ± 0.86 | 1.40 ± 1.60 | <0.001 |  | 1.25 ± 1.52 | 1.40 ± 1.61 | 0.2697 |
| ALB(g/dL) | 4.08 ± 0.38 | 3.79 ± 0.48 | <0.001 |  | 3.92 ± 0.55 | 3.81 ± 0.47 | 0.0112 |
| GLB(g/dL) | 7.05 ± 0.58 | 6.90 ± 0.67 | <0.001 |  | 6.94 ± 0.69 | 6.91 ± 0.68 | 0.6194 |
| FBG (mg/dl) | 106.60 ± 38.29 | 111.33 ± 41.03 | 0.063 |  | 105.87 ± 40.25 | 110.78 ± 40.66 | 0.1651 |
| INR | 1.05 ± 0.30 | 1.07 ± 0.42 | 0.218 |  | 1.09 ± 0.39 | 1.07 ± 0.43 | 0.5877 |
| APTT (ms) | 31.01 ± 5.29 | 31.34 ± 4.67 | 0.326 |  | 31.55 ± 7.10 | 31.33 ± 4.57 | 0.6703 |
| BMI (kg/m^2^) | 23.62 ± 3.24 | 22.91 ± 3.28 | <0.001 |  | 23.41 ± 3.27 | 22.97 ± 3.28 | 0.1272 |
| HGB(g/L) | 136.30 ± 18.92 | 129.02 ± 22.96 | <0.001 |  | 130.35 ± 22.55 | 130.04 ± 22.64 | 0.8726 |
| mRS score at admission | 0.64 ± 1.27 | 0.70 ± 1.35 | 0.490 |  | 0.71 ± 1.34 | 0.70 ± 1.35 | 0.818 |
| SEX |  |  | 0.890 |  |  |  | 0.5924 |
| Men | 969 (61.33%) | 165 (60.89%) |  |  | 154 (58.8) | 161 (61.5) |  |
| Women | 611 (38.67%) | 106 (39.11%) |  |  | 108 (41.2) | 101 (38.5) |  |
| Age(years) |  |  | 0.273 |  |  |  | 0.6308 |
| <60 | 371 (23.48%) | 55 (20.30%) |  |  | 63 (24) | 55 (21) |  |
| 60-70 | 421 (26.65%) | 66 (24.35%) |  |  | 67 (25.6) | 64 (24.4) |  |
| 70-80 | 552 (34.94%) | 99 (36.53%) |  |  | 92 (35.1) | 93 (35.5) |  |
| ≥80 | 236 (14.94%) | 51 (18.82%) |  |  | 40 (15.3) | 50 (19.1) |  |
| Hypertension | 998 (63.16%) | 170 (62.73%) | 0.891 |  | 154 (58.8) | 163 (62.2) | 0.4747 |
| Diabetes | 492 (31.14%) | 98 (36.16%) | 0.101 |  | 77 (29.4) | 94 (35.9) | 0.1360 |
| Smoking | 624 (39.49%) | 107 (39.48%) | 0.997 |  | 104 (39.7) | 104 (39.7) | 1.0000 |
| AF | 336 (21.27%) | 63 (23.25%) | 0.464 |  | 57 (21.8) | 62 (23.7) | 0.6766 |

Values are mean ± standard deviation or median (quartile) or number (%)

TC, total cholesterol; PLT, platelet; HGB, hemoglobin concentration; LDL-c, low-density lipoproteins cholesterol; GLB, globulin; HDL-c, high-density lipoprotein cholesterol; blood urea nitrogen; Scr, serum creatinine; BUN, TG, triglyceride; FBG, fasting blood glucose; ALB, serum albumin; BMI, body mass index; mRS, modified Rankin scale; AF,Atrial fibrillation; INR,international normalized ratio.

**Table S4. Factors influencing 3-month poor functional outcome by univariate logistic regression analysis.**

|  | Statistics | OUTCOME |
| --- | --- | --- |
| HDL-c(mg/dl) | 47.012 ± 13.523 | 0.999 (0.991, 1.006) 0.736 |
| BMI (kg/m^2^) | 23.517 ± 3.255 | 0.920 (0.891, 0.951) <0.001 |
| HGB（g/dL） | 13.524 ± 1.973 | 0.820 (0.778, 0.863) <0.001 |
| TG (mg/dl) | 110.704 ± 57.484 | 0.997 (0.995, 0.999) <0.001 |
| LDL-c(mg/dl) | 107.928 ± 37.412 | 0.997 (0.994, 0.999) 0.019 |
| ALB(g/dL) | 4.035 ± 0.412 | 0.262 (0.202, 0.340) <0.001 |
| GLB |  |  |
| BUN (mg/dl) | 17.481 ± 8.560 | 1.019 (1.007, 1.030) 0.001 |
| Scr (mg/dl) | 1.078 ± 1.012 | 1.030 (0.936, 1.134) 0.543 |
| mRS score at admission | 0.652 ± 1.282 | 1.501 (1.391, 1.619) <0.001 |
| Sex |  |  |
| Male | 1134 (61.264%) | Ref |
| Female | 717 (38.736%) | 1.664 (1.356, 2.043) <0.001 |
| Diabetes |  |  |
| No | 1261 (68.125%) | Ref |
| Yes | 590 (31.875%) | 1.437 (1.162, 1.776) <0.001 |
| Hypertension |  |  |
| No | 683 (36.899%) | Ref |
| Yes | 1168 (63.101%) | 1.371 (1.107, 1.699) 0.004 |
| PLT（10*109/L） | 222.612 ± 70.055 | 0.999 (0.998, 1.001) 0.288 |
| Age(years) |  |  |
| <60 | 426 (23.015%) | Ref |
| 60-70 | 487 (26.310%) | 1.139 (0.824, 1.575) 0.431 |
| 70-80 | 651 (35.170%) | 1.860 (1.388, 2.493) <0.001 |
| ≥80 | 287 (15.505%) | 3.995 (2.861, 5.580) <0.001 |
| Smoking |  |  |
| No | 1120 (60.508%) | Ref |
| Yes | 731 (39.492%) | 0.613 (0.495, 0.759) <0.001 |
| AF |  |  |
| No | 1452 (78.444%) | Ref |
| Yes | 399 (21.556%) | 2.015 (1.597, 2.542) <0.001 |
| FBG (mg/dl) | 107.295 ± 38.730 | 1.009 (1.006, 1.011) <0.001 |
| Fibrinogen(g/L) | 3.282 ± 0.728 | 1.446 (1.261, 1.659) <0.001 |

Values are mean ± standard deviation or median (quartile) or number (%)

HGB, hemoglobin concentration; PLT, platelet; TC, total cholesterol; TG, triglyceride; LDL-c, low-density lipoproteins cholesterol; HDL-c, high-density lipoprotein cholesterol; Scr, serum creatinine; BUN, blood urea nitrogen; GLB, globulin; ALB, serum albumin; BMI, body mass index; FBG, fasting blood glucose; CHD, coronary heart disease; mRS, modified Rankin scale;

**Table S5. Relationship between** fibrinogen **and mRS score 3-month after acute ischemic stroke**

| Exposure | Non-AIS（n= 1325） | AIS(n= 526) | Model (β 95%CI)  *p* |
| --- | --- | --- | --- |
| Fibrinogen (g/L) | 3.23 ± 0.68 | 3.43 ± 0.83 | 0.163 (0.058, 0.268) 0.002 |
| Fibrinogen quartiles |  |  |  |
| Q1 | 348 (26.26%) | 111 (21.10%) | 0 |
| Q1 | 348 (26.26%) | 112 (21.29%) | 0.063 (-0.144, 0.270) 0.552 |
| Q3 | 331 (24.98%) | 128 (24.33%) | 0.148 (-0.061, 0.358) 0.165 |
| Q4 | 298 (22.49%) | 175 (33.27%) | 0.261 (0.048, 0.473) 0.016 |
| P for trend |  |  | <0.001 |

we adjusted age, BMI, sex, LDL-c, TG, HGB, HDL-c, BUN, FPG, ALB, PLT, AF, hypertension, smoking, DM, mRs score at admission.

β, beta coefficient.

Table S6 Relationship between fibrinogen and unfavorable outcome 3-month after **acute ischemic stroke** in different sensitivity analyses.

| Exposure | Model I (OR 95%CI) P | Model I (OR 95%CI) P |
| --- | --- | --- |
| Fibrinogen (g/L) | 1.213 (1.029, 1.430) 0.021 | 1.253 (1.046, 1.502) 0.015 |
| Fibrinogen quartile |  |  |
| Q1 | 1.0 | Ref |
| Q2 | 1.000 (0.710, 1.409) 0.998 | 0.847 (0.569, 1.261) 0.414 |
| Q3 | 1.138 (0.810, 1.600) 0.455 | 1.313 (0.894, 1.929) 0.164 |
| Q4 | 1.336 (0.949, 1.880) 0.097 | 1.373 (0.937, 2.011) 0.104 |

Model I was a sensitivity analysis in participants without TG ≥200mg/dL(n=1717). We adjusted age, sex, BMI, LDL-c, HGB, HDL-c, BUN, FPG, ALB, PLT, AF, hypertension, smoking, DM, mRs score at admission.

Model II was a sensitivity analysis conducted on BMI<25kg/m^2^ participants (n= 1302). we adjusted age, sex, LDL-c, HGB, TG, HDL-c, BUN, FBG, ALB, PLT, AF, hypertension, smoking, DM, mRs score at admission.

OR, odds ratios; CI, confidence; Ref: reference.

Table S7 Association between fibrinogen and unfavorable outcome at 3 months after acute ischaemic stroke after propensity score matching

| Exposure | OR (95%C) P |
| --- | --- |
|  |  |
| low fibrinogen(<4.0g/L) | Ref |
| High fibrinogen(≥4.0g/L) | 1.681 (1.171, 2.412) 0.005 |

OR, odds ratios; CI, confidence; Ref: reference.

Table S8. Stratified associations between fibrinogen and 3-month unfavorable outcomes in patients with AIS by age, sex, hypertension, smoking status, and diabetes.

| Characteristic | No. of patients | Effect size(95%CI) | P value | P for interaction |
| --- | --- | --- | --- | --- |
| Sex |  |  |  | 0.571 |
| Male | 1134 | 1.196 (0.977, 1.465) | 0.083 |  |
| Female | 717 | 1.303 (1.033, 1.644) | 0.025 |  |
| Age (years) |  |  |  | 0.541 |
| <60 | 426 | 1.331 (0.954, 1.857) | 0.093 |  |
| 60-70 | 487 | 1.002 (0.731, 1.375) | 0.988 |  |
| 70-80 | 651 | 1.317 (1.026, 1.690) | 0.026 |  |
| ≥80 | 287 | 1.235 (0.873, 1.748) | 0.232 |  |
| Hypertension |  |  |  | 0.849 |
| 683 | 683 | 1.224 (0.962, 1.557) | 0.100 |  |
| 1168 | 1168 | 1.252 (1.026, 1.528) | 0.027 |  |
| Diabetes |  |  |  | 0.546 |
| No | 1261 | 1.201 (0.994, 1.453) | 0.059 |  |
| Yes | 590 | 1.322 (1.019, 1.715) | 0.036 |  |
| Smoking |  |  |  | 0.566 |
| No | 1120 | 1.203 (0.994, 1.455) | 0.057 |  |
| Yes | 731 | 1.316 (1.018, 1.701) | 0.036 |  |

Note 1: Above model adjusted for age, sex, BMI, LDL-c, HGB, TG, HDL-c, BUN, FPG, ALB, PLT, AF, hypertension, smoking, DM, mRs score at admission.

Note 2: In each case, the model is not adjusted for the stratification variable

OR, odds ratios; CI, confidence;


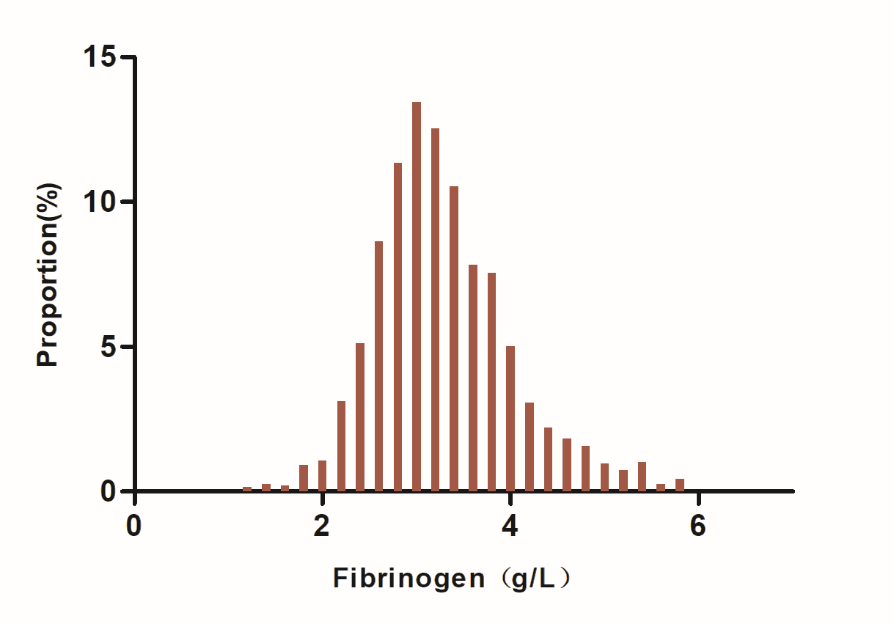


**Figure S1. Distribution of fibrinogen.**

Figure S1. It presented a normal distribution, ranging from 1.25 to 5.85 g/L with a mean ± standard deviation of 3.282 ± 0.782g/L


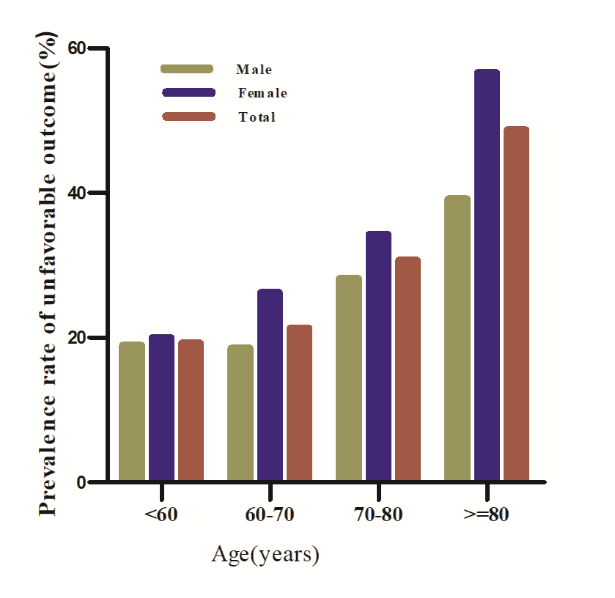


**Figure S2. The incidence rate for unfavorable outcomes after acute ischemic stroke of age stratification by 10 intervals.**

Figure S2 showed that the incidence of unfavorable outcomes among participants with AIS was higher in women than in men, regardless of age group. Incidence unfavorable outcomes were also found to increase with age in both males and females.
